# Applying an environmental public health lens to the industrialization of food animal production in ten low- and middle-income countries

**DOI:** 10.1186/s12992-019-0479-5

**Published:** 2019-06-13

**Authors:** Yukyan Lam, Jillian P. Fry, Keeve E. Nachman

**Affiliations:** 1 Johns Hopkins Center for a Livable Future, Johns Hopkins Bloomberg School of Public Health, 111 Market Place, Suite 840, Baltimore, MD 21202 USA; 2 Department of Environmental Health & Engineering, Johns Hopkins Bloomberg School of Public Health, 615 N. Wolfe St., Baltimore, MD 21205 USA; 3 Department of Health, Behavior and Society, Johns Hopkins Bloomberg School of Public Health, 624 N. Broadway, Baltimore, MD 21205 USA; 4 Department of Health Policy and Management, Johns Hopkins Bloomberg School of Public Health, 624 N. Broadway, Baltimore, MD 21205 USA; 5 Risk Sciences and Public Policy Institute, Johns Hopkins Bloomberg School of Public Health, 615 N. Wolfe St., W7007, Baltimore, MD 21205 USA

**Keywords:** Industrial food animal production, Lower and middle-income countries, Meat, Animal feed, Land use, Policy analysis

## Abstract

**Background:**

Industrial food animal production (IFAP) is characterized by dense animal housing, high throughput, specialization, vertical integration, and corporate consolidation. Research in high-income countries has documented impacts on public health, the environment, and animal welfare. IFAP is proliferating in some low- and middle-income countries (LMICs), where increased consumption of animal-source foods has occurred alongside rising incomes and efforts to address undernutrition. However, in these countries IFAP’s negative externalities could be amplified by inadequate infrastructure and resources to document issues and implement controls.

**Methods:**

Using UN FAOSTAT data, we selected ten LMICs where food animal production is expanding and assessed patterns of IFAP growth. We conducted a mixed methods review to explore factors affecting growth, evidence of impacts, and information gaps; we searched several databases for sources in English, Spanish, and Portuguese. Data were extracted from 450+ sources, comprising peer-reviewed literature, government documents, NGO reports, and news articles.

**Results:**

In the selected LMICs, not only has livestock production increased, but the nature of expansion appears to have involved industrialized methods, to varying extents based on species and location. Expansion was promoted in some countries by explicit government policies. Animal densities, corporate structure, and pharmaceutical reliance in some areas mirrored conditions found in high-income countries. There were many reported weaknesses in regulation and capacity for enforcement surrounding production and animal welfare. Global trade increasingly influences movement of and access to inputs such as feed. There was a nascent, compelling body of scientific literature documenting IFAP’s negative environmental and public health externalities in some countries.

**Conclusions:**

LMICs may be attracted to IFAP for economic development and food security, as well as the potential for increasing access to animal-source foods and the role these foods can play in alleviating undernutrition. IFAP, however, is resource intensive. Industrialized production methods likely result in serious negative public health, environmental, and animal welfare impacts in LMICs. To our knowledge, this is the first systematic effort to assess IFAP trends through an environmental public health lens for a relatively large group of LMICs. It contributes to the literature by outlining urgent research priorities aimed at informing national and international decisions about the future of food animal production and efforts to tackle global undernutrition.

## Background

As appetites for animal products increase globally, the practice of industrial food animal production (IFAP) has expanded in many parts of the world. This mode of production is characterized by high throughput methods that involve housing thousands to millions of animals per year in close proximity at a single location [1]. Waste management at these facilities is typically handled in a manner that leads to unhygienic production conditions. In the case of chickens raised for meat, dry manure from production is only partially removed between flocks; for other species, waste is typically handled in liquid form and temporarily stored in manure lagoons before being applied to agricultural land as fertilizer, sometimes exceeding appropriate agronomic rates [2–5]. The manure harbors a variety of microbiological and chemical agents, many of which can pose health risks when released into the environment, especially among persons who may encounter them in air, water or soil, or who may ingest them when consuming animal products [6–15]. These production methods often rely on an array of veterinary pharmaceuticals, including antibiotics from classes important to human medicine, and arsenic-based drugs [16–18]. The use of some of these compounds has been shown to select for bacteria that can cause antibiotic resistant infections in exposed persons.

While often purported to be an efficient means of producing animal protein, evidence exists to suggest that IFAP is responsible for significant public health and ecological burdens. In part due to the inputs required, but also due to the magnitude of waste produced by this system, IFAP facilities are responsible for the generation and release of myriad environmental health hazards that have been demonstrated to impact the health of animal house workers, community residents, persons consuming animal products derived from this system, and the global ecosystem. Evidence suggests that the industrialized model of animal production also can have serious consequences for the welfare of animals raised within this system [1, 19–22].

Occupational exposures faced by employees of IFAP facilities are well documented, as workers have the most contact with the hazards generated by the production system. Numerous studies have documented increased risks of respiratory disease [11, 23–27], as well as colonization with antimicrobial-resistant bacteria among animal house workers [28–31].

Hazards generated on-site have also been shown to leave the property of the production facility and expose persons residing on adjacent properties and in surrounding communities [8, 32, 33]. These releases can occur as a result of ventilation fans used to moderate the temperature of the facility and through the discharge of animal wastes, both through direct discharge and through management practices used to transport wastes away from production facilities and onto agricultural fields [5, 34]. Animal transport trucks and other non-domesticated vectors like flies have been shown to transport antibiotic- resistant bacteria into communities [35, 36]. Studies of members of communities proximate to IFAP have found significant associations with respiratory disease, antibiotic-resistant bacterial carriage and infection, mental health outcomes, and other adverse health conditions [7, 8, 12–15, 37–43].

The production of animals is resource intensive, and requires large quantities of feed and water [44]. Beyond the direct impacts felt by those working in and living near IFAP facilities, research has documented the global impact of feed and water provisioning for animal production, and how it may contribute to worsening climate and water availability crises [45–47].

The industrialized method of animal production was first developed in the United States [1], and following its establishment, similar production operations have become commonplace in other high-income countries, supporting diets that are relatively high in animal-based food as compared to the rest of the world [48]. In many lower- and middle-income countries (LMICs), however, animal-based food consumption levels are low, mainly due to lack of availability and poverty; as incomes rise in these countries a corresponding increase has been observed in appetites for animal-based foods [48, 49].

Increased consumption of animal-based foods is an important component of efforts to address the burden of undernutrition globally. Stunting and other forms of undernutrition are still widespread and remain a leading cause of high morbidity and mortality in LMICs [50]. In 2017, the global prevalence of stunting was 22.2%, with regional prevalences of 23.2% for Asia, 9.6% for Latin America and the Caribbean, and 30.3% for Africa [50]. Further, stunting has irreversible effects, in particular on cognitive development, with repercussions on childhood school performance and economic productivity in adult life, as well as on maternal and neonatal mortality and morbidity when stunted children themselves bear children [51]. Children who are undernourished in their first 2 years of life also face higher risk of chronic diseases when they experience rapid weight gain in later childhood or adulthood [52]. Higher intake of animal-source foods in the first 1,000 days of life is an essential strategy for alleviating these burdens [53, 54].

Given this challenge, LMICs may indeed be interested in increasing the availability of animal-source foods by increasing domestic food animal production. Prior evidence suggests that adoption of industrialized production methods is increasing in LMICs [55], occurring alongside the global expansion of western-owned food animal production companies [56]. Further, research has shown that rising disposable incomes are associated with enhanced dietary diversity, including the addition of animal-source foods [57]. Considering the negative externalities described above, the reliance on industrialized methods in LMICs for increasing the availability of animal-source foods raises concerns because people living in LMICs already bear the largest burden of morbidity and mortality caused by environmental contamination, in part due to a lack of resources for pollution control infrastructure and effective regulatory controls [58]. The purpose of this paper is to assess the current state of IFAP growth in a relatively large and geographically diverse set of LMICs, with special attention on the environmental public health impacts that may be unintended consequences of this trend. Our mixed methods review describes features of IFAP growth in these countries, identifies factors enabling or inhibiting industry expansion, synthesizes evidence of impacts, and characterizes key information gaps.

## Methods

The research methodology followed three main steps: country selection, literature search, and data extraction. Methods, described in brief below, are elaborated in greater detail elsewhere [59].

### Country selection

We used a series of analytical steps and metrics to select ten low- and middle-income countries that provided geographic variability. First, we excluded high-income countries, based on World Bank designations, and countries with less than one million inhabitants. Then, based on each country’s livestock numbers as reported by the United Nations Food and Agriculture Organization (FAO) from 2013, we calculated the total animal units (AUs) for cattle, chickens, and pigs, using conversion factors previously applied by the US EPA. We also calculated AUs per agricultural area to derive AU density. Based on the metrics of total AUs and AU density, we selected Ethiopia, Myanmar, India, and Vietnam. Based on total AUs, we selected Brazil and China. Finally, considering species- specific production, total AUs, and the goal of achieving reasonable geographic coverage, we added Mexico, Turkey, Kenya, and Uganda.

### Literature search

A mixed methods review was used to identify i) peer- reviewed literature, ii) non-peer reviewed sources (i.e., report from a non-governmental organization or government agency), and iii) news articles; this approach combines review methods and source types to provide a broader understanding of a topic than a review based solely on the peer-reviewed literature [60]. We conducted searches for information in English, Portuguese, and Spanish in 2015. The date restrictions that we applied varied by source, but in general we restricted to sources published from 2000 onward. We searched for peer-reviewed academic articles and non-peer reviewed reports using PubMed (no date restriction), Scopus, the US Department of Agriculture’s Agricultural Online Access (AGRICOLA) database, Google Scholar, and Google. Specific search terms, by source/database, are listed in the appendix. For news articles, we searched Google News (restricted to 2010 onward), Global Meat News, Feed Navigator, and Environmental Health News—Above the Fold (no date restriction for these latter sources).

The goal was to balance breadth and depth across and within countries to the extent possible. Thus, for countries yielding less information, we looked further back in time for references. For countries yielding numerous recent references, we cut off the search at more recent dates, after obtaining adequate references to cover the topics in sufficient depth. In addition to source language (English, Portuguese, or Spanish) and publication date (as described above), additional inclusion criteria were that there be clear authorship (including institutional authors) and subject matter relevance. Personal blogs and opinion pieces were excluded. Overall, we cast a wide net in our search strategy to ensure that we could capture sufficient information about an evolving situation, for which there has been limited attention and resources for research.

### Data extraction and analysis

Of the documents collected using the search strategy described above, approximately 10% were excluded upon further examination because, notwithstanding their titles/abstracts, they lacked relevant information or were insufficiently reliable or comprehensible. Data were extracted from the remaining references using a template that contained the following categories: basic information about the document; a summary; numerical projections on food animal production; livestock industry characteristics; impacts of IFAP; public engagement with food animal production issues; and comments about the quality of the reference. In total, information was extracted from over 450 documents. The number and type of documents are shown by country in Table 1.

**Table 1:** Sources for data extraction

Country	Journal articles	News articles	Others (e.g., NGO/IGO reports, conference papers, government sources)
Brazil	26	27	20
China	43	42	18
Ethiopia	28	1	14
India	15	16	19
Kenya	13	3	5
Mexico	10	7	6
Myanmar	5	2	5
Turkey	20	2	12
Uganda	14	11	16
Vietnam	23	9	32

Information was analyzed first by country, and then synthesized by theme, following the above-mentioned categories. The quality of the documents was taken into account during synthesis in that we relied more heavily on sources in the peer-reviewed scientific literature, those issued by intergovernmental, government, and non- governmental organizations, and those that were relatively recent.

## Results

Results from reviewing the country sources are synthesized below by topic. We start with describing the policy and regulatory landscape around food animal production, and then outline general features and notable trends regarding specific aspects of production. These range from industry characteristics to production practices and inputs to IFAP impacts.

### Domestic policies

#### Policies driving IFAP in these LMICs

We found consistent evidence that domestic policies have played an important role in the industrialization of food animal production in low- and middle-income countries. Policies include facilitating access to production inputs and services, providing technical assistance, subsidized credit, low-interest loans, tax breaks, and other forms of financial assistance, reducing trade barriers, strengthening private property rights, land leases, and land reform. Sources referenced such policies in Brazil [61], China [62–66], India [67, 68], Mexico [69–72], Turkey [73–75], and Vietnam [76–83].

The benefits provided are often available only to production units operating at a minimum scale. This can result in a “distorting” of the market to the detriment of smaller-scale producers [67]. For example, in China, the official designation of “dragonhead enterprise” is given to companies meeting a certain scale of production, technology use, and management, and enables those companies to receive subsidies and tax breaks from various levels of government and confers greater legitimacy [64, 66]. Other examples include the state of Uttar Pradesh in India, where subsidies of up to $830,000 US dollars were provided to farms with at least 10,000 parent units of broiler chickens [68], and the East and Southeast Annatolia regions of Turkey, where the government covered 30% of construction and 40% of breeding equipment costs for new cattle stock farms containing at least 50 heads [75, 84].

Moreover, the policies often fit within overarching national livestock development strategies that promote expansion, vertical integration, consolidation, and intensification of food animal production. In China, the country’s twelfth “Five-Year Food Industry Plan (2010-2015)” envisioned a 50% reduction in small- scale pork slaughterhouses by 2015 through mergers and acquisitions [85]. Vietnam’s 2020 national livestock development plan promotes industrialization and integration, and aims for large- scale intensive livestock farms to produce 70% of the country’s meat, and for industrial slaughtering to account for approximately 35% of the meat supply by 2020 [76].

In the region of sub-Saharan Africa, the national governments of Ethiopia, Uganda, and Kenya also encourage expansion of livestock production; however, it appears emphasis has been on rural development and improved livelihoods instead of scale or industrialization [86–89]. In Ethiopia, the government’s “National Livestock Development Projects” focus on small-scale operations that increase household income through ‘improved’ livestock rearing, rather than on increased output [86]. In Uganda, the “National Agriculture Policy” launched by the president in 2014 specifies, as part of its six objectives, increased food security, farming household income, and human resources [89]. In these contexts, government and non-governmental actors have provided extension services, training, and other support to help farmers increase productivity and efficiency [87, 88, 90–92].

#### Regulations

Another major theme emerging from our review is concern over insufficient or inadequate regulation of food animal production in low- and middle-income countries experiencing IFAP expansion. One principal domain of concern is the oversight of inputs used in raising livestock, including antimicrobial drugs and other growth- promoting compounds, which has implications for public health and food safety. Despite some recognition of the problem and government efforts to address it, ineffective monitoring and/or inadequate regulations have persisted as challenges in countries like China [93–95], Ethiopia [96], India [97], Myanmar [98], Uganda [92], and Vietnam [76, 83, 99–101].

Another key domain pertains to the environmental impacts of food animal production activities, including wastewater discharge, manure management, and disposal of dead animals. Various sources attest that regulations in this domain have been difficult to enforce, too lax, or absent altogether in Brazil [67, 102], China [103–105], India [67], Kenya [106], Mexico [107], Myanmar [83], and Turkey [108–110]. For example, two studies in Turkey documented non-compliance with environmental regulations regarding storage of animal waste [109, 110], while a study in Mexico noted a rise in confined animal feeding operations—“CAFOs” —but no standard definition of a “CAFO” or regulations on minimum distances that these operations have to be set back from residential areas [107]. According to a comparative legal study, animal agriculture in Brazil is often exempted from animal and environmental laws, which have weak enforcement mechanisms, and an official estimate of 40% of animal farms operate informally, and therefore outside the sphere of regulation [111].

A few sources also highlighted weak regulations or inadequate enforcement of regulations to protect animal welfare, specifically in Brazil [111, 112], China [105], and Turkey [113]. For example, some standards on animal welfare in Brazil are only voluntary [112], while another norm that penalizes animal cruelty does not cover slaughter [111].

Difficulties in regulating food animal production were sometimes ascribed to inefficient inter-institutional collaboration [83, 93, 100], reliance on local government enforcers [114, 115], and disparate standards enacted across country regions [102]. However, the regulatory landscape was described as evolving in countries like China, where three new laws came into effect in 2015 aimed at addressing food safety, environmental impacts, and advertising and labeling [116].

### Industry characteristics

#### Larger, more profit-oriented operations

Across the countries studied, the upsurge of larger commercial operations raising only one type of livestock animal has occurred alongside a decline in smaller subsistence- oriented farms over the past few decades. We found many sources that sought to classify a facility’s scale of production based on the type and number of live animals raised at any given time or per season/year. Despite this tendency, the thresholds are not uniform across countries. For example, a typology for broiler production in Brazil deems “small” producers to be those with less than 10,000 broilers and “large” producers to be those with 10,000 broilers or more [67]. Meanwhile, in Vietnam, units with up to 2,000 broilers are considered “small-” or “medium-scale,” and those with 2,000 or more are considered “intensive” and “large-scale” [117]. There is a tendency to divide animal operations by scale and apply labels to them, but caution should be taken in interpreting these labels, since they are dependent on context and may even vary with time. For this reason, we refrain from providing a uniform typology here, and instead refer readers to country-specific sources with classifications for Brazil [67], China [63, 65, 66, 103, 118, 119], Ethiopia [120–122], India [123], Kenya [90, 123, 124], Mexico [69, 70], Turkey [108], Uganda [92, 125, 126], and Vietnam [76, 127, 128].

In any case, most live animals or meat production in a given sector in the countries we researched can still be attributed to smaller-scale operations [76, 92, 100, 120–122, 124–126, 128–132]. However, the role of larger-scale operations is expanding [86, 103, 113, 126, 131–137]. In China, for example, large-scale pig farms raising 3,000 to 50,000 pigs per year were responsible for 16% of the country’s pigs by 2010, while medium-scale and small-scale farms raised 48% and 34%, respectively [103]. In Vietnam, intensification and scaling up are reflected in the declining percentage of pigs raised on farms with no more than ten pigs—from 80% in 1999 to 64% in 2006 [131].

In addition, larger-scale or more intensive operations tend to be found in specific geographic areas of a given country. In Ethiopia, poultry farms with capacity for 10,000 birds have been established in urban areas around and east of the country’s capital, and account for one to two percentage points of national production [120, 122, 129]. Similarly, more intensive meat production is based in urban and peri-urban areas in Uganda, while extensive systems of subsistence-based production continue to be more prevalent in that country’s arid and semi-arid regions [138]. In Vietnam, poultry production is well developed everywhere, but is especially concentrated in urban areas and the Red River and Mekong River deltas [128, 130, 139].

#### Concentration

Source documents described some tendency toward concentration of food animal production or related sectors (such as feed or meat processing) in several countries studied [66, 70, 90, 92, 118, 119, 124, 125, 140–148]. The scope of this study did not include mapping corporate structure or subsidiary relationships. Nevertheless, it does appear that IFAP expansion has been fueled by a mix of entities, including US-based corporations and foreign-based corporations that have adopted a production model similar to that of their US counterparts. Some of these foreign entities operate in multiple countries, while others are focused in a specific LMIC. In Brazil’s Southern Region, which started experiencing industrialization of the pork and poultry industries several decades ago, the number of large-scale broiler farms increased by 67% between 1974 and 1992, while the number of broiler farms overall decreased by 24% [143]. In China, consolidation of the pig sector has resulted in family farmers choosing between exiting the sector, becoming specialized hog producers, or working as waged, migrant laborers [66]. In Mexico, one source described the poultry sector as exhibiting more concentration than the United States, with three producers accounting for 60% of the market by 2005 [72]. According to another source, structural readjustment imposed by the International Monetary Fund (IMF) and economic liberalization coincided with 27% of poultry producers leaving the business between 1980 and 1990 [70].

The poultry sector is also where concentration is most evident in Turkey, and most production is attributed to integrated enterprises using contract farming [141, 149]. By the mid-2000s, the top 20 and top five of 66 integrated broiler companies accounted for 84% and 47% of the market share, respectively [145]. Researchers have described this level of concentration as dampening competition, and rendering the broiler sector a loose oligopoly [141].

Concentration has also been documented in related sectors, such as animal feed and meat processing. In the Brazilian state of Rio Grande do Sul, the top five pork and poultry processing companies controlled 63% and 85% of their respective markets in 2009 [142]. The Kenyan vertical integrator, Farmer’s Choice, is characterized by the FAO as having a monopoly on pork processing, handling 80% of the pigs processed in the country [90], while one Ugandan company is reportedly responsible for 85% of Kampala’s processed meat market and holds a monopoly over beef processing [125]. In Vietnam, a news source reported that Masan Group is undertaking acquisitions and expansion, with the goal of attaining 50% of the country’s feed market by 2020 [148].

#### Vertical integration

In a few of the countries studied, sources documented a substantial amount of vertically integrated production and processing, and the presence of large integrating firms [66, 69, 70, 84, 100, 127, 130, 145, 150–153]. In Brazil, over 90% of poultry production occurred within vertically integrated systems by 2012 [154]. In China, at least 70% of pork and poultry production and 80% of aquaculture production operate through the most highly integrated and government- endorsed form of vertical integration, in which integrating companies are designated officially as “dragonhead enterprises” and receive special benefits [151]. Vertical integration has been described as increasing across Asia [155], with much of the poultry in India and Vietnam now being raised by contract farmers producing for vertical integrators [134, 153, 156].

In other countries, such as Ethiopia and Kenya, the extent of vertical integration has been much less, with at most one or two companies in each livestock sector operating as integrators [87, 106, 124, 157]. Despite the small number of integrators, integrated production appears to be on the rise in the Kenyan poultry sector [90], while official and non-governmental sources in Ethiopia are arguing for increasing vertical linkages and supply chain development [86, 129, 158, 159].

Sources for some countries, such as Brazil, Mexico and Turkey, noted that producers seeking to continue production independently have been unable to compete with vertically integrated producers, who can source inputs from integrators, reduce their transaction costs, and reap economies of scale [67, 72, 141, 160]. A few studies have found that integrated farmers sometimes face lower profit margins or inequitable contracts [142, 153, 160, 161].

### Feed

Feed is one of the costliest inputs to food animal production, and has been identified as one of the most important determinants behind the expansion of IFAP [67, 84, 162–165]. For many countries, the scarcity of feed and resources for producing feed has impeded IFAP expansion. In China, feed production has grown to become a multibillion-dollar industry [166], but there remains a shortage of water, land, and labor, to produce sufficient feed grains domestically, such as corn and soy [167]. Intensification of animal agriculture in China is exacerbating the feed shortage [114], as well as incentivizing cultivation practices like high-density planting, monoculture growing, and mechanization [167]. Within Brazil, certain regions are able to produce large quantities of corn and soy, and these crops are transported over long distances to regions where animal production is concentrated [168]. The cattle sector in Mexico has coped with a lack of grazing land and feed resources by exporting live cattle to be finished elsewhere [169]. Turkey and Vietnam have increased industrial feed production, but they rely heavily on feed crop imports [74, 100, 165, 170]. Vietnam spent an estimated 4.5 billion USD on imports of feed materials (mostly corn and soybean) in 2014 [171]. Foreign feed companies have a dominant role in Vietnam’s feed production [100, 172], and some of these companies work with integrated farms that raise animals intensively [152]. Meanwhile, in Uganda, feed produced domestically is reported to be very poor quality. Problems include low nutrient content, mixing with non-feed materials to increase the weight of the feed, feeds that have been moistened, and feeds containing toxins harmful to humans (as well as animals), such as aflatoxin [173, 174]. In contrast to these countries, Myanmar is described as being self-sufficient in livestock feed thus far, though it does import feed supplements and additives [83].

One specific issue noted in Ethiopia, India, and Kenya pertains to the opportunity cost of using scarce resources to produce grains for animal feed, in a context of food insecurity: such resources can be used more efficiently to produce crops directly consumed by humans [88, 124, 164, 175–177]. In Ethiopia, the government aims to increase annual domestic feed production from 5 million kg in the early 2010s to 14.5 million kg by 2025 [178]. Currently, most grain produced in the country is used to feed humans, and one NGO has warned that using domestically-produced grain to feed livestock animals rather than humans may threaten food security in Ethiopia, especially if these animals are subsequently exported [175]. Expanding grain production is a challenge, given the scarcity of arable land and problems with over-grazing and degraded pastures [179].

### Antimicrobials and other growth-promoting compounds

In most of the studied countries, there is indication that antimicrobials and other growth-promoting compounds are being used in livestock production with inadequate veterinary oversight. Notable examples include China, where a large, uncertain quantity of such additives is used for both prophylactic and growth promoting purposes [105, 115, 180–187], and India, where non-therapeutic usage of antibiotics by the poultry industry has become such a problem that the Indian Medical Association has demanded measures to prevent medically important antibiotics from being used [188]. Other examples are described in Ethiopia [175, 189, 190], Kenya [88, 106], Mexico [169, 191], Myanmar [83, 98], Uganda [192–196], and Vietnam [99, 101, 197]. The extent of the problem and information available varies by country and sector, and generally more problems have been documented in the pig and poultry sectors.

Across studies and countries, information about the use of antimicrobials and other growth promoting compounds was presented inconsistently, precluding quantitative estimation of use. By antimicrobial class, we found most frequent mention of tetracyclines (in five countries), followed by aminoglycosides, beta-lactams and macrolides, each in three countries. Arsenicals, fluoroquinolones, ionophores, penicillins, polymixins (colistin), polypeptides, and sulfonamides were mentioned as being used in two or fewer countries. We also found mention of beta-agonists ractopamine (in China) and clenbuterol (in China and Mexico). By species, mention of antimicrobial use was most common in chicken production (six countries), followed by swine (four countries) and cattle (three countries).

### Animal welfare (housing facilities)

Information was limited on the welfare of livestock animals, especially regarding the physical conditions in which they are raised. Sources for a few countries noted the level of confinement and density of livestock housing. For example, in Brazil, poultry are reportedly raised in a high degree of confinement [154], at an average density of 34 kg per square meter, according to an industry report [198]. Animals in China, especially poultry and swine, are also raised in extremely confined and densely crowded conditions [119, 187, 199]. Even small- and medium-sized chicken farms located in the more remote parts of the country stock birds at a high density, and use antibiotics as inputs to counter their reduced immunity [119, 187]. One study in Ethiopia documented a density of 13 birds per square meter for a 10,000-broiler farm, which was described as having poor biosecurity and hygiene practices [200], while another source noted that overcrowding prevented animals from expressing their natural behaviors in cattle feedlots and confined dairy cow facilities, as well as caged poultry systems [175]. In India, poultry cages added to farms after 2012 were required to be a minimum of 750 square centimeters, a 50% increase from the prior standard [201]; however, a 2008 source noted that most of the country’s poultry flock was raised in open houses, and only in winter in a few regions were birds housed indoors in heated shelters [202].

International animal welfare NGOs have noted concerns in Brazil’s swine farms, such as the use of gestation crates, mutilations, very early weaning age, limited bedding, close confinement of sows, and insufficient climate control for young piglets [203, 204]. Researchers described a lack of regulations on broiler and swine housing conditions, such as no norms on ammonia concentrations, temperatures, heat stress risk, and noise level exposure [198]. In China, non-governmental organizations have also documented the common use of gestation crates for swine and battery cages for layers and broilers, with both practices being perceived as Western and ‘scientific’ [95, 187]. Ventilation, especially in broiler farms and beef cattle sheds, is poor, while floors tend to be made of hard concrete, causing lameness in cattle and preventing pigs from rooting [187].

### Slaughtering and processing infrastructure

Slaughtering and processing capacities vary greatly across the ten selected countries. In some cases, there has been an expansion of infrastructure apace with or even outstripping the supply of live animals, while in other countries, slaughtering and processing are undertaken mostly by small- and medium-scale operators with poor hygiene and outdated facilities. In the Chinese pork and beef sectors, processing capacity has exceeded the supply of live animals [65, 205]. Modernized slaughterhouses receiving preferential treatment from the government in the form of tax breaks, low-interest loans, and other assistance have operated below capacity [65]. In Mexico, the number of federally-inspected plants, considered the most sophisticated slaughterhouses, more than quadrupled between 1999 and 2005, but operated at 55 to 60% capacity as of 2000 [69]. Similarly, one source described Uganda’s slaughtering facilities as operating at 50% capacity due to the lack of live animals [125]. At the same time, the three main slaughterhouses serving Kampala are considered overburdened, and meat-processing has been essentially monopolized by one integrating firm controlling 85% of the capital’s processed meat market [125].

Sources in several other countries also highlighted inadequate infrastructure. For example, in India, processing was described as unhygienic [206], and a top priority of the government’s Five-Year Plan from 2012 to 2017 was to upgrade the country’s registered meat-processing plants and export-oriented slaughterhouses [164]. In Kenya, a 2014 report noted that 30% of broilers were slaughtered in large- or medium-scale slaughterhouses; the rest were slaughtered in rudimentary, on-farm facilities [90]. As for swine, one integrator dominated the landscape, with a factory that slaughtered 400 pigs daily as of 2012; in contrast, the other three main slaughterhouses in Kenya slaughtered only 15 to 50 pigs daily [90]. In the Vietnamese swine sector, a 2008 survey revealed that of 434 slaughterhouses, only 45% had licenses to operate, 35% had sanitary facilities, and 25% had running water [100].

Overall, however, it appears that many countries have at least some industrial-scale facilities with ‘modern’ equipment and high throughput, as well as plans to build more of these facilities [65, 69, 76, 84, 100, 139, 207–209]. For example, alongside backyard slaughtering and thousands of slaughter points (facilities with very small daily slaughtering capacities), Vietnam has 35 industrial-scale slaughterhouses, most of which are located in the Red River Delta [76], and the government is actively promoting the expansion and upgrading of slaughterhouses [100].

### Land use

In a few of the countries studied, food animal production has triggered controversies over land use, given the limited arable land available in certain countries. In Ethiopia, rural communities in the Gambela region and Lower Omo Valley have denounced “land-grabbing” and displacement by investors, including multinational corporations, who are establishing industrialized agricultural enterprises in the region [210]. The amount of land in the country is said to be insufficient for sustaining current levels of food animal production, let alone increases in production [175]. In Uganda, the government itself has warned that increasing livestock numbers will put greater pressure on rangelands and water resources [91]. The Chinese government has urged companies, especially large-scale DHEs, to invest in overseas land and feed deals as part of the country’s overall strategy to expand livestock production [151]. Feed destined for China has been sourced from Africa, Eastern Europe, Southeast Asia, and Latin America [167]. Chinese companies have established soybean contract farming in Brazil [211], and various other land acquisition deals for soy production are also in development in Argentina [167].

Land scarcity has been used as a justification for more intensive food animal production practices. The swine sector in China is said to focus increasingly on landless industrial systems that source feed externally and do not have any land for manure disposal (manure is rarely recycled given the lack of surrounding land) [103]. CAFOs have been portrayed as the only way to support rising demand for meat, given the country’s limited land base [151]. In Uganda, a government document pointed to free-range extensive production systems as an inefficient use of land and a source of resource management conflicts in the Albertine Rift region [212]; others have suggested intensifying production and adopting zero-grazing systems in overstocked areas [213]. There are also proponents of zero-grazing systems in Ethiopia, who argue that confining animals in feedlots can prevent over-grazing and environmental degradation [213]. A similar argument—that intensification can help reduce deforestation—has also been presented in Brazil, and the issue has received significant attention because of the public sentiment against conversion of the Brazilian Amazon into cattle ranches. Various sources in Brazil, including ones tied to the government, have attempted to show that the increase in cattle production is due to intensification, rather than deforestation and expansion of pastures [214–220]. However, there has been some recognition that intensification may lead to more deforestation over the long run, if the sector appears more attractive [61].

### Waste management

For various countries, like Brazil [221], China [222], and Vietnam [223], the amount of manure and wastewater generated by food animal production is an environmental and public health concern because the sheer volume of waste is difficult to manage. An estimated 1.9 billion tons and 227 million tons of manure excretion and pollution, respectively, resulted from all livestock production in China in 2010, corresponding to 1.86 tons of livestock manure pollution per hectare of arable land in the country [222]. Under the scenario of business as usual, total livestock manure pollution is projected to increase 31% to 298 million tons by 2020 [222]. Swine waste, in particular, accounts for 47% of total livestock waste generated in China [85].

Sources referred to waste management practices in varying detail. Some sources described practices for treating or disposing of animal waste, but this information may not have been exhaustive, and it is not always known how widely certain methods are practiced. Although incomplete, the general picture emerging from various countries is that waste is not being adequately treated prior to discharge [83, 87, 90, 100, 103, 104, 108–110, 124, 135, 180, 224, 225].

Specific practices in Brazil include storage of waste in uncovered, open slurry tanks [168] and application of liquid manure to land [102, 168, 221]. In China, animal waste from large-scale facilities is separated into solid parts—which are dried and sold as fertilizer or used as compost—and liquid parts—which are occasionally stored in open-air lagoons or diluted with large quantities of water to be used for irrigation [104]. A couple sources noted issues with liquid manure spilling or leaching into surrounding soil, rivers, and other water bodies [103, 180]. Although some large swine farms have received subsidies from the government to build biogas tanks to manage waste, distribution and utilization of huge volumes of biogas slurry and residue persist as challenges [85]. In Vietnam, waste is also separated into solid and liquid parts, followed by composting of solid parts and discharge of liquid parts into crop fields and surface water, with little pretreatment [82, 225]. Even in industrial-scale facilities, there has been a lack of waste treatment capacity and awareness of laws on managing waste [83].

In countries with relatively less intensive production, lower livestock numbers, and hence smaller volumes of waste, waste management is still problematic. In Kenya, manure disposal has been identified by the FAO as a major challenge for commercial pig farms that are not integrated with crop production, as some of these farms dump manure onto the roadside, on uncultivated land, or into sewage and storm water drains, causing both air and water pollution [124]. Poultry farms, on the other hand, sell manure without first composing it, and this may be a source of disease for other farms where the manure is applied to the land [87]. In Turkey, cattle-fattening enterprises reportedly dump manure onto unoccupied areas [108], or apply it as fertilizer without any treatment [109]. Most farms store waste in open areas by villages or barns for months at a time, or sometimes even indefinitely [109, 110]. Although a few cattle farms have a manure storage hole, the holes may be poorly built [109].

Inadequate or patchy regulation of animal manure management and disposal of dead animals was noted in Brazil [67, 102]. In Mexico, one 2010 source indicated that swine CAFOs in one area have opted to pay fines rather than invest in expensive wastewater treatment infrastructure [226]. A study of 135 cattle farms in western Turkey revealed that most farms have ignored regulations that limit the storage of animal waste to three months, with up to half storing waste for six to eight months [109]. Further, regulations on the distance required between water resources and waste storage facilities were not being followed [110].

### International trade as related to LMICs

International trade considerations are important for a few of the countries studied, but in rather different ways. For example, China meets its growing demand for animal feed not only by importing feed crops, such as whole soybeans [166], but also by investing in contract farming abroad [211]. There are also reports that China is sourcing or intends to source live animals from Australia and Mongolia [227, 228]. Brazil, on the other hand, is an exporter of beef, and its trade relationships have been influential in production practices within the country. For example, it has committed to not using growth promoters in animal products exported to Russia [229, 230], and concerns about the use of ractopamine has resulted in Russia banning Brazilian pork and beef imports at various times [229–231]. Similarly, Canada banned meat from two poultry plants in Brazil for several years when it detected antibiotic residues [232]. Brazilian meatpacking companies have also responded to pressure from the EU market to regulate the use of antibiotics, given the importance of that market for Brazilian exports [233].

Mexico’s defining trade relationship is with the United States, due to geographic proximity and the North American Free Trade Agreement (NAFTA), which is said to have driven the industrialization of the country’s livestock sector [234]. Following NAFTA, multinational agribusinesses acquired a dominant role in Mexico, obtaining competitive advantages over domestic companies because they could obtain large volumes of feed much cheaper in the United States and could import them tariff-free [70, 234].

Ethiopia faces yet a different problem, with illegal cross-border trade in cattle assuming a significant role: an estimated 320,000 heads of cattle are exported illegally each year (a figure from the mid-2000s) [235]. This activity, typically undertaken by small-scale traders, reportedly hinders beef cattle production and value chains by creating a shortage of live animals and processed meat for legal export [235].

### IFAP impacts and lack of impact studies

Overall, the impacts of industrialized food animal production in the selected LMICs have not been thoroughly examined. There are several domains of concern, including occupational health, environmental health, and other socio-economic and community impacts. However, most sources mentioned these concerns in a general way; in the peer-reviewed literature, there have been relatively few primary research studies investigating a given issue or site.

There is variation by country and topic regarding the extent to which impacts have been studied or even mentioned. For example, our search methodology yielded little research and attention regarding worker health across all of the countries. As for environmental and public health, concerns about impacts caused by industrialized food animal production were noted in many of the countries studied, and a few countries even had primary research on these issues.

Reports from Vietnam, for instance, flagged concerns about antibiotic resistance associated with the use of commercial poultry feed [236], bad odors and increased zoonotic disease risk caused by industrial livestock farms near urban areas [128], reduced biodiversity due to extinction of indigenous breeds [237], atmospheric pollution and water depletion caused by the industrialization of livestock production [76], contamination of surface and groundwater from concentrated swine production [131], and generation of more animal waste than could be recycled naturally due to expanded livestock production, with associated threats to soil, water, and air quality, as well as public health [76].

In Brazil, several studies noted that intensified cattle ranching has attracted more producers into the market and fueled deforestation, overgrazing, greenhouse gas emission, and use of herbicides, fungicides, and insecticides [61, 217, 220, 235]. However, it was also claimed that methane emissions per unit product has decreased, deemed a sign of greater efficiency [217, 220, 235]. Comparative assessments noted that industrial systems in Brazil rely on feed with a larger water footprint [238], and linked the production of animal feed for intensive livestock farming to greenhouse gas emissions, loss of biodiversity, freshwater eutrophication, marine eutrophication, and terrestrial acidification, among other impacts [168].

In Mexico, there have been resident reports of respiratory health issues due to hog pollution in the Perote Valley area [226], and governmental investigations found decreased aquifer levels, increased odors, and poor air quality due to swine farms in that region [224]. Meanwhile, another study documented a high concentration of antibiotic-resistant pathogens near an urban-based industrial dairy operation in northern Mexico [107].

In Turkey, sources alluded to water and air pollution from industrial scale animal farming [108] and the livestock sector’s significant contributions to methane emissions [239]. One study found that cattle breeding operations in one region were inadequately handling animal waste, leading to odors, flies, and contamination of soil and water resources [110].

There were also many sources on China, some involving empirical investigations and others consisting of secondary data. Specific concerns included infectious diseases due to inadequately treated animal waste [104, 240], drug resistance emerging from the overuse of antibiotics in animal production [93, 180, 182, 184, 186, 241], cancer risk deriving from trace metals, like arsenic, in manure [104, 240], severe food poisoning from the use of a steroid that promotes lean muscle growth [105], high levels of methane emissions from manure, which are more than any other country [242, 243], and pollution of surrounding land and water, with waste as a primary source of contamination [104, 151, 166, 240]. According to one review article, larger farms cause more serious environmental contamination [115], and another report noted that methane emissions from industrial farms are higher due to the manner in which waste is stored and handled [242]. However, within policy circles in China, many argue that industrialization of livestock production is beneficial for the environment because more concentrated waste management will lead to more precise techniques being used, greater investment in facilities, and easier monitoring [244].

Two studies sought to characterize drug resistance in pathogens isolated from livestock fecal samples in Uganda. One looked across chickens, cattle, swine, and small ruminants [192], and the other, focused on broiler farms with greater than 100 chickens [194]; both found multidrug resistance in most isolates obtained. Another source mentioned the pressure of increasing livestock numbers in the country on rangelands and water systems [91].

We found less information about environmental and public health impacts in the other countries studied. There were brief references to concerns about contamination from waste and decomposing carcasses in India [67], disease transmission risk from poultry manure [87] and water and air pollution from swine manure in Kenya [124].

Finally, socioeconomic impacts due to industrialization of the livestock sector have also been examined by sources from several countries. The disparity between larger-scale producers and smaller-scale producers has been a particular concern, especially when the latter can no longer remain competitive. For example, research in Brazil documented that larger-scale swine and broiler producers cause more environmental harm than smaller-scale counterparts, but smaller farms internalize environmental costs more than larger ones [67]. In Vietnam, a 2003 study cited increased unemployment and impoverishment due to smaller producers leaving the sector and fewer rural employment opportunities as possible negative effects of intensified production [237]. Sources in China noted that in the context of agricultural industrialization, smallholders are losing their livelihood, inequality is increasing, and rural-to-urban migration is on the rise [166]. The pork industry’s consolidation has caused small-scale farmers to choose between becoming specialized producers or waged migrant workers [66]. Similarly, in Turkey, researchers have argued that governmental initiatives promoting the expansion of the livestock sector have been harmful to smallholder producers, who have been unable to stay competitive in the new policy and economic environment [73, 245].

With vertical integration a key feature of industrialization, several studies have examined whether contract farming can be a mechanism for small-scale producers to maintain their livelihoods. An investigation of poultry farming in one county of Kenya showed that contract farming was associated with a significant, positive gain in net revenue per bird, among a sample of 180 small-scale poultry farms [246]. In India, however, a 2007 report reviewing the literature on the impact of contract farming on smallholders concluded that contract farming could help them stay in business, but that there was conflicting data on whether contract or non-contract farming was more profitable for farmers and limited evidence showing that vertical coordination could reduce risk and transaction costs for small farmers [202]. A subsequent study on integrated broiler production in India found that small-scale farmers tended to be excluded, contract farmers perceived contract terms to be unfair, and contract farmers ultimately reverted back to being independent producers after obtaining experience and achieving a certain scale of production [153].

## Discussion

The sources we reviewed indicate increased uptake of the IFAP model in the selected LMICs regarding size of operations, corporate consolidation, and vertical integration. These trends vary by species and geography, though IFAP is not the dominant production model in many of the countries. Smaller and independent producers in some regions have either exited the sector or specialized and expanded in size, in a context of shifts to larger operations, more control by fewer companies, and use of contract growers. In addition, corporate consolidation is seen in feed and meat processing, so small and/or independent businesses at multiple stages of the supply chain may be impacted. Uncertainty remains regarding the costs and benefits of losing independent actors in the supply chain, and whether these changes improve producers’ livelihoods and in-country development goals more broadly. An important consideration is the availability of alternative livelihoods for small and independent animal and feed producers and processors, especially if there is a large number leaving the sector in one region.

### Domestic policies

Many of the examined countries have explicit plans to industrialize, while others (such as the African countries studied) are more focused on improving the livelihoods of small-scale farmers. In countries seeking to industrialize food animal production, governments have instituted policies favoring large operations that use certain production methods, and increased centralized capacity in related sectors like slaughtering and meat processing. Many of the LMICs studied currently have a combination of smaller, older slaughterhouses and processing facilities and large, newer facilities that might operate at half capacity because the domestic supply of animals does not match the size of the facilities. Our review found that governments often play key roles in supporting and promoting new processing facilities in pursuit of the IFAP model.

### Access to land and feed

Animal production is resource-intensive compared to production of plant-based foods, in large part due to the land required to feed animals [247]. While animals and their feed are co-located in grazing systems, industrially- produced animals can be separated from where feed crops are grown. This separation between confined animals and feed cropland has led to promotion of IFAP as an efficient way to produce animal products compared to extensive/grazing systems, especially where land is scarce. Land is required to produce feed crops, however, and some countries and corporations are establishing relationships abroad to ensure land is available to produce feed for domestic animal production. Among other potential negative impacts, global expansion of land used to produce animal feed can lead to deforestation, which threatens biodiversity and contributes to global climate change [248, 249].

Access to feed inputs has been a significant impediment to industrialization in certain countries, stemming from a lack of resources to produce feed within those countries and/or an inability to import feed. Where feed is produced domestically, quality concerns have been documented. If there is a significant increase in global feed production, it is possible that industrialization will occur more rapidly as that barrier is removed. There is evidence of concern in some countries that meeting demand for feed through domestic production will threaten food security because less grain will be available for direct human consumption. It is unclear whether there are safeguards in any countries to control the competition for resources between human food and animal feed.

### Global trade

As industrialization of the food animal sector progresses in the studied countries, our review suggests that trade will play an increasingly important role for these countries in the context of our globalized food system. In particular, trading of feed inputs [250] and animal stock [251], as well as the leasing and foreign ownership of land [252], all connect domestic food production to other parts of the globe. This trading acquires heightened importance considering the natural resource scarcities observed in various LMICs studied. In some cases, existing international trade agreements may facilitate exchange of inputs and export of finished products across adjacent borders. Additionally, food safety considerations of high-income countries related to the use of pharmaceuticals in animal production [253] may erect short-term trade barriers for countries’ export markets, which may ultimately influence future usage patterns of these compounds (as observed in Brazil’s trading relationships with Russia and Canada).

### Dietary patterns and resource use

As noted previously, consumption of animal-based foods in LMICs, especially among the most food-insecure, could have a positive impact on undernutrition and its impacts. The issue at stake, however, is how to increase availability of and access to the appropriate animal- source foods sustainably and without creating new public health threats. Though not the focus of this paper, an additional concern is the disease risk associated with over-consumption of animal-based and processed foods, as populations of LMICs adopt a more “western-style” diet [254].

An important concept related to feeding a growing and more affluent global population in the coming years is sustainable intensification (SI), which refers to producing more food while reducing use of resources and limiting environmental impacts [49]. A major focus of SI, although no specific agreed-upon definition exists, is increasing crop yields on underperforming fields, or closing yield gaps. There are many points of contention among various food system and agricultural experts over whether the focus should be on increasing crop yields given potential negative externalities (e.g., increasing use and runoff of commercial fertilizers, and better yields with no rise or a reduction in farmers’ incomes due to costs of inputs) [255]. Regarding production of animal protein, some see the promise of SI as facilitating increased feed production without using more arable land by closing yield gaps, but rapid growth in demand for feed could eclipse yield gains and fail to reduce overall land and water use. If strategies incorporating SI into IFAP are too narrowly focused, efforts by private and public stakeholders to address the serious environmental and public health impacts associated with expansion of IFAP will not be successful.

### Infrastructure and regulations for protection of environmental health

There is a nascent, yet compelling, body of scientific literature on the impacts of IFAP in some of the studied countries, which reinforces concerns regarding negative environmental and public health externalities, especially when IFAP operations are located close to urban/peri-urban and other populated areas. This is consistent with research demonstrating similar problems in high- income countries, even in the face of arguably more regulations and monitoring infrastructure [8]. Moreover, although there are gaps in the information we could find for specific countries and certain topics, such as occupational health, there has been evidence of these and other impacts in the United States. We did not find evidence that these circumstances would be meaningfully different in LMICs. As regulatory frameworks, monitoring and enforcement capacity, and technical expertise (e.g., in veterinary health) are less developed in LMICs, there is reason to believe that impacts would be worse overall—a hypothesis that underscores the need for research and policy attention to IFAP issues in LMICs.

For example, our literature review did not identify stringent regulations concerning control of pharmaceutical agents and delivery of veterinary medical services in the context of IFAP. There is fierce debate over the effectiveness of these regulatory measures on antimicrobial resistance in high income countries [253]. Despite this, weak veterinary infrastructures and ready access to these drugs were repeatedly mentioned as concerns in the studied countries and have been identified as public health concerns in the literature [256]. Similarly, regulation of waste management is especially critical in countries that currently have established IFAP due to the volume of excrement and mortalities requiring disposal. In the LMICs studied, while some animal waste is used on fields as fertilizer, there are issues due to a lack of waste storage infrastructure required to properly compost wastes before use, indiscriminate disposal in places with potential public contact, and noncompliance where regulations do exist. It is uncertain how broadly these poor waste management practices apply to sectors in each country. On the issue of occupational health, the lack of information on worker well-being in our selected LMICs likely reflects the challenges of underreporting and conducting research with these hard-to-reach study populations (e.g., migrant or undocumented workers, job-insecure and economically vulnerable laborers). The challenges, documented elsewhere [257–261], are likely exacerbated in low-resource settings.

### Animal welfare

Lastly, in addition to environmental and public health impacts, industrialization of food animal production in LMICs may have important implications for animal welfare globally. Regulatory protections for animal welfare have been described as weak in the United States [18]. Similarly, we did not find evidence of comprehensive regulatory protection of animal welfare in the examined countries. Though availability of information on the conditions in which animals are kept was limited, it appears that in LMICs where IFAP has developed, animal housing conditions are similar to those of industrial operations in the U.S. and other high-income countries, especially regarding density. Research has highlighted the interconnections between compromised animal welfare and public health concerns. For example, increased stocking density has been demonstrated to contribute to the transmission of pathogens among animals, to workers and into surrounding communities [7, 38, 262]. Moreover, animals confined in crowded conditions are not able to express natural behaviors [19], which can lead to stress and poor animal health. Civil society campaigns based on examinations of animal welfare within IFAP operations have been successful at incrementally improving conditions in some high-income countries (e.g. [204]). It is unclear whether similar efforts would be effective in the LMICs we examined.

### Limitations

While our review of available peer-reviewed and gray literature yielded insights into the changing nature of food animal production in LMICs, it is important to acknowledge its limitations. We identified a disproportionate number of peer reviewed articles and other sources among countries. For example, the available literature concerning IFAP in China and Vietnam was quite substantial, while far fewer sources were available concerning production in Myanmar. While this may reflect a true differing degree of research and media coverage of animal production practices across countries, it may also be related to language barriers (our searches of the literature were limited to sources written in English, Portuguese, or Spanish). It is likely that as production expands in these countries, more information will become available regarding the factors evaluated in this manuscript. In the meantime, it would be a productive endeavor to examine native-language documents describing animal production in each of the countries included in our review, especially to have a fuller picture of domestic regulatory landscapes.

Moreover, given our objective of scoping an emerging situation, we were fairly inclusive in the types of sources relied upon, which extended beyond peer-reviewed scientific literature. The different types of “grey literature” sources used most certainly varied in quality. There was no formal method for assessing and weighing sources based on quality, which we recognize as a limitation of this study. Nevertheless, we hope our findings stimulate further research—particularly research conducted by or with in-country partners—to elucidate the issues set forth here.

## Conclusion

To our knowledge, this is the first systematic effort to assess IFAP trends through an environmental public health lens for a relatively large group of LMICs. We have identified existing or potential negative externalities of this mode of production in LMICs, as many of these impacts are foreshadowed by the experience of high- income countries where IFAP currently predominates. There is an urgent need for site-specific research to elucidate the impacts of IFAP on public health, the environment, the livelihoods of producers operating at varying scales, and animal welfare. This research agenda should also seek to identify case studies of successful, sustainable modes of food animal production in an LMIC, which could reveal lessons and best practices relevant for other producers, regions, and countries.

The question of whether IFAP growth facilitates food security globally or nationally also warrants special attention. For countries that are struggling with undernutrition and its severe, long-term impacts on health and well- being, we recognize that increasing availability of certain animal- source foods can be part of the solution and may alleviate national food insecurity. However, in addition to output metrics, the characteristics of production and distribution must also be considered. For example, how can increased availability of animal-source foods be best directed toward key vulnerable populations, such as children experiencing chronic malnutrition? Further, if increasing production does alleviate food insecurity, can such gains be sustained, given natural resource constraints? The relationship between increased IFAP and food security is complex, and will need to be investigated at the national, regional, and global scales.

From production to consumption of animal-source foods, the most appropriate strategies for change will likely vary across different places. In this vein, future assessments would benefit from the involvement of in-country partners. The findings from our mixed methods review contribute to the literature by outlining urgent collaborative research priorities aimed at informing national- and international-level decisions about the future of food animal production.
